# Learning to Predict miRNA-mRNA Interactions from AGO CLIP Sequencing and CLASH Data

**DOI:** 10.1371/journal.pcbi.1005026

**Published:** 2016-07-20

**Authors:** Yuheng Lu, Christina S. Leslie

**Affiliations:** Computational Biology Program, Memorial Sloan Kettering Cancer Center, New York, New York, United States of America; Rutgers University, UNITED STATES

## Abstract

Recent technologies like AGO CLIP sequencing and CLASH enable direct transcriptome-wide identification of AGO binding and miRNA target sites, but the most widely used miRNA target prediction algorithms do not exploit these data. Here we use discriminative learning on AGO CLIP and CLASH interactions to train a novel miRNA target prediction model. Our method combines two SVM classifiers, one to predict miRNA-mRNA duplexes and a second to learn a binding model of AGO’s local UTR sequence preferences and positional bias in 3’UTR isoforms. The duplex SVM model enables the prediction of non-canonical target sites and more accurately resolves miRNA interactions from AGO CLIP data than previous methods. The binding model is trained using a multi-task strategy to learn context-specific and common AGO sequence preferences. The duplex and common AGO binding models together outperform existing miRNA target prediction algorithms on held-out binding data. Open source code is available at https://bitbucket.org/leslielab/chimiric.

## Author Summary

MicroRNAs (or miRNAs) are a family of small RNA molecules that guide Argonaute (AGO) to specific target sites within mRNAs and regulate numerous biological processes in normal cells and in disease. Despite years of research, the principles of miRNA targeting are incompletely understood, and computational miRNA target prediction methods still achieve only modest performance. Most previous target prediction work has been based on indirect measurements of miRNA regulation, such as mRNA expression changes upon miRNA perturbation, without mapping actual binding sites, which limits accuracy and precludes discovery of more subtle miRNA targeting rules. The recent introduction of CLIP (UV crosslinking followed by immunoprecipitation) sequencing technologies enables direct identification of interactions between miRNAs and mRNAs. However, the data generated from these assays has not been fully exploited in target prediction. Here, we present a model to predict miRNA-mRNA interactions solely based on their sequences, using new technologies to map AGO and miRNA binding interactions with machine learning techniques. Our algorithm produces more accurate predictions than state-of-the-art methods based on indirect measurements. Moreover, interpretation of the learned model reveals novel features of miRNA-mRNA interactions, including potential cooperativity with specific RNA-binding proteins.

## Introduction

Recent high-throughput technologies like AGO CLIP sequencing [1, 2] and CLASH (crosslinking, ligation, and sequencing of miRNA-RNA hybrids [3]) enable direct biochemical identification of AGO binding and miRNA target sites transcriptome-wide. The miRNA field has a strong tradition of computationally leveraging transcriptome-wide data to improve target site prediction, but the leading miRNA target prediction methods today do not exploit these new biochemical data. Here we present a systematic approach to learn both the rules of miRNA-target site pairing and a binding model of AGO’s local sequence preferences and positional bias in alternative 3’UTR isoforms in order to accurately predict miRNA-target interactions.

Before it became possible to map AGO-mRNA and miRNA-mRNA interactions directly, the major advance in miRNA target prediction came from restricting to predefined classes of miRNA seed matches in 3’UTRs and training a model to predict mRNA expression changes in miRNA overexpression experiments. TargetScan was the first algorithm to introduce the strategy of correlating context features of miRNA seed sites—including flanking AU content, position in the 3’UTR, and complementarity to the 3’ end of the miRNA—with extent of target down-regulation in miRNA transfection experiments [4]. Similar observations were encapsulated in the TargetRank method [5], and these studies established that rules of miRNA targeting could be statistically decoded from transcriptome-wide data.

However, new data from AGO CLIP sequencing and CLASH challenge some of the assumptions of existing prediction strategies. These data confirm the prevalence of non-canonical target sites lacking complementarity to the miRNA 2–7 (6-mer) seed region and conversely show that even exact miRNA 2–8 (7-mer) seed matches are often not AGO bound [6, 7]. Meanwhile, most target prediction methods require strong seeds to avoid false positives. For example, downloadable predictions from the most recent version of TargetScan still require either perfect 2–8 seed complementarity (7-mer-m8 site) or a 2–7 seed with A across from miRNA position 1 (7-mer-1A site), although AGO CLIP data suggests that 7-mer and 8-mer seeds are found in only about half of AGO binding sites [6]. The mirSVR method [8], which also trains on miRNA overexpression experiments, allows up to one mismatch or G:U wobble in the 6-mer seed region, but in practice few non-canonical sites are assigned even moderate scores. Therefore, current target prediction methods may focus on detecting the most effective miRNA sites at the cost of missing a large proportion of miRNA-mRNA interactions. Furthermore, training on non-physiological miRNA overexpression experiments may obscure more subtle targeting rules.

A few studies have developed algorithms to resolve which highly expressed miRNAs are associated with individual AGO CLIP peaks. For example, microMUMMIE is an algorithm for analysis of AGO PAR-CLIP that uses the location of T-to-C mutations—indicative of the site of cross-linking of the RNA-binding protein to the RNA in the PAR-CLIP assay—to assign the most likely canonical seed [9]. Other methods use energy-based duplex prediction to associate miRNAs with CLIP-mapped target sequences [10–13]. In particular, MIRZA uses an unsupervised probabilistic approach to learn parameters of a duplex alignment model from AGO CLIP peaks, and the duplex model can be used to make *de novo* miRNA target site predictions from 3’UTR sequence [12]. Note that the MIRZA study used the term “non-canonical” to refer to sites lacking 7 or 8 nucleotides of perfect complementarity to the 5’ end of the miRNA; therefore, their reported non-canonical sites included both perfect 6-mer and many 7-mer-1A sites. (We will use “non-canonical” exclusively for sites lacking full complementarity in the 2–7 6-mer seed region.) More recently, MIRZA-G combined MIRZA duplex quality scores with known context features like flanking AU content and predicted secondary structure accessibility as well as conservation, once again to predict extent of down-regulation in miRNA overexpression experiments [14].

Here we present a novel model for miRNA target prediction through discriminative learning on transcriptome-wide AGO CLIP and CLASH profiles. Our goal was to learn to accurately predict biochemical miRNA-target site interactions, rather than the extent of regulation, in order to increase the sensitivity of miRNA target prediction and learn physiological targeting rules. As the CLASH protocol captures direct interactions between miRNAs and mRNAs by ligation, it provides a partially labeled training set of miRNA-mRNA interactions including many non-canonical pairings, which we combined with canonical AGO binding sites identified by CLIP. We trained one support vector machine (SVM) classifier to model the miRNA-mRNA duplexes and a second SVM to learn AGO’s local sequence preferences in the UTR and positional bias in 3’UTR isoforms. The duplex SVM model enables the prediction of both canonical and non-canonical pairings between miRNA and target sequences and outperforms existing methods for assignment of miRNAs to AGO binding sites. The AGO binding model is trained using a multi-task strategy to distinguish between cell type and protocol specific sequence signals and common AGO sequence preferences. The duplex SVM and common AGO binding SVM together outperform existing target prediction approaches when evaluated on held out interaction data. Our prediction method, called chimiRic, is available as open source code at https://bitbucket.org/leslielab/chimiric.

## Results

### ChimiRic learns both miRNA-mRNA duplex structures and AGO binding preferences from CLIP and CLASH data

**Fig 1:**
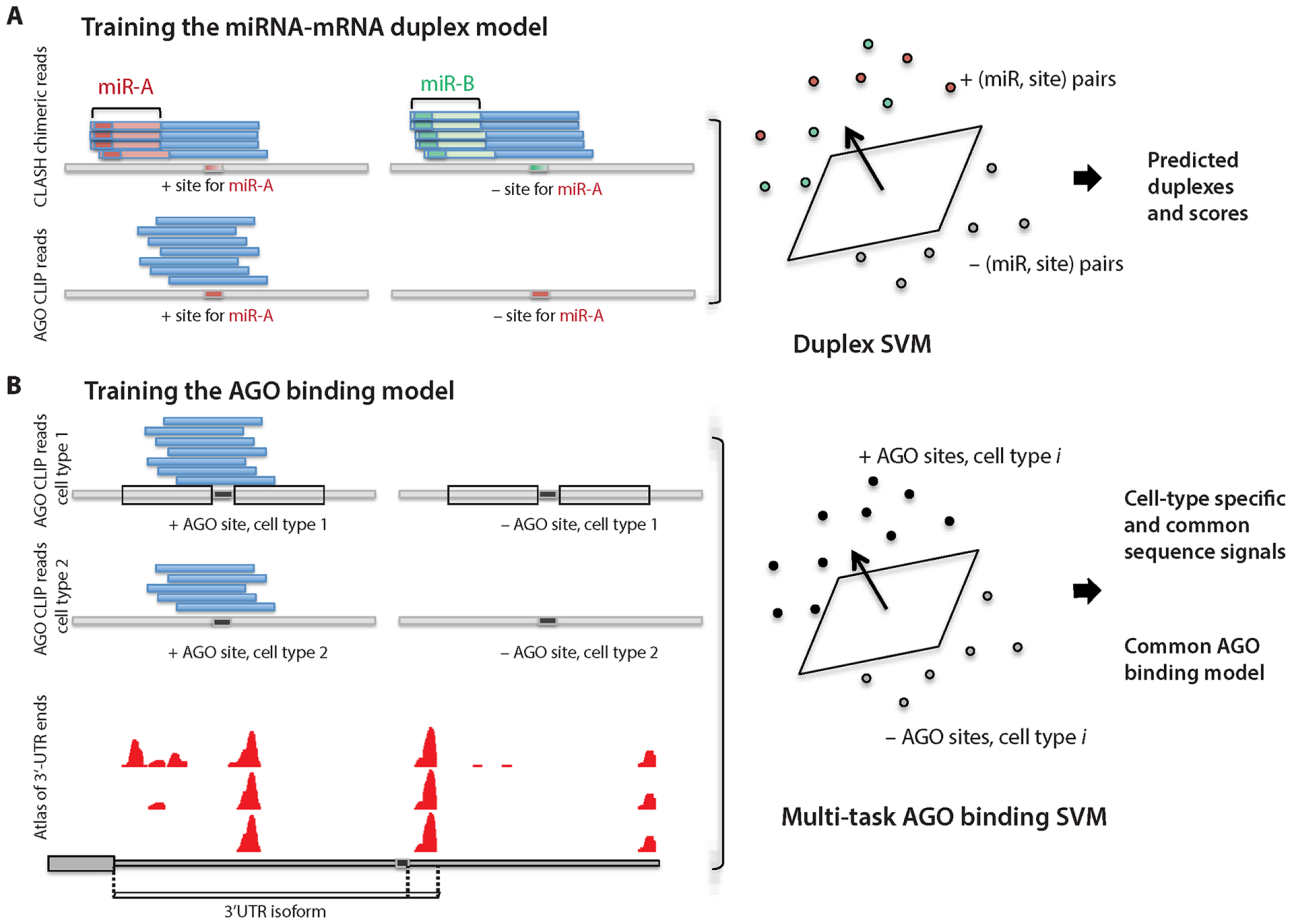
Overview of the chimiRic prediction model. (**A**) The first component of the chimiRic model is the duplex SVM, which learns to predict and score miRNA-mRNA duplex alignments from CLASH and CLIP-seq data. Positive (miRNA, site) training examples comprise canonical and non-canonical pairings identified by chimeric reads in CLASH data (top left) as well as sites with canonical miRNA seeds supported by AGO CLIP data (bottom left). Negative (miRNA, site) training examples include sites that are paired with a different miRNA based on CLASH chimeric read data (top right) or miRNA seed matches with no AGO CLIP evidence (bottom right). The duplex SVM learns the parameters for local duplex sequence alignment and predicts optimal alignments for (miRNA, site) pairs through an iterative training procedure (see Materials and Methods). (**B**) The second component of chimiRic is the AGO binding SVM, which uses features encoding the positional bias of AGO binding sites relative to (possibly multiple) 3’ ends of transcripts as well as the local positional *k*-mer sequence features. Mouse and human ApA atlases based on 3’ end sequencing data (bottom) provide the coordinates of 3’ ends used in the analysis.

ChimiRic’s duplex model is trained on chimeric reads from CLASH data, which associates a miRNA with a target sequence via chimeric reads and can identify non-canonical binding sites, and AGO CLIP binding sites containing a 6-mer seed match (or longer seed) for a single highly expressed miRNA (Fig 1A). In the latter case, differential AGO CLIP-seq analysis suggests that an AGO bound site that can be associated with a unique miRNA by a canonical 6-mer seed is likely a binding site for that miRNA [6].

We used CLASH [3] and AGO PAR-CLIP data [2, 15] in HEK293 cells to train the duplex model, restricting to the top 59 expressed miRNAs in 21 miRNA seed families (S1 Table, Materials and Methods). To compile the training set, sites identified by CLASH chimeric reads were required to fall within 3’UTRs, contain a sequence within an edit distance of 1 (substitutions or indels) from a canonical 6-mer seed match for the interacting miRNA, and also be supported by non-chimeric reads (see Materials and Methods). This filtering yielded the *positive* training examples consisting of 1,727 (miRNA, site) pairs supported by chimeric reads, of which 1,228 were non-canonical interactions, together with 11,211 canonical (miRNA, site) examples from AGO CLIP sites (Fig 1A). Canonical miRNA seed matches that are not AGO bound based on CLIP data, together with (miRNA, site) pairs where an AGO-bound site is paired with an incorrect miRNA, provided 25,411 *negative* examples. To compensate for the class imbalance, we only used a randomly sampled subset of negative examples in training (see Materials and Methods).

We trained a structural SVM [16] on positive and negative (miRNA, site) training examples to learn a model for predicting miRNA-site duplex alignments. Here, the model vector ***w*** of the SVM represents the scoring parameters for local pairwise alignment. SVM training proceeds iteratively, alternating between obtaining optimal alignments of all training examples given the current SVM parameters ***w*** and updating the model vector ***w*** given the current duplex alignments (Materials and Methods, S1 Fig). The model update step involves solving the SVM large-margin optimization problem so that the discriminant scores assigned to positive and negative (miRNA, site) examples have the correct sign and obey margin constraints, with a hinge loss function to control margin violations (see Materials and Methods). To define the local alignment scoring system and convert the alignment score into an SVM discriminant function, we used a parameterization similar to the energy-based scoring system in MIRZA, namely a match/mismatch score that depends on the position in the miRNA sequence together with the nucleotides being aligned and penalties for loop opening and for symmetric and asymmetric loop extensions (see Materials and Methods). One important difference with MIRZA is that the chimiRic alignment can only start at position 1 of the miRNA if is it matched against nucleotide A, which more accurately reflects known determinants of miRNA targeting [17].

The second component of chimiRic’s scoring system is an SVM classifier that learns to discriminate the local sequence features and positional bias in 3’UTR isoforms of true AGO binding sites versus sites that contain 6-mer seed matches of highly expressed miRNAs but are not AGO-bound, as determined by CLIP data (Fig 1B). Here we considered two AGO CLIP sequencing data sets, the human HEK293 PAR-CLIP data set [2] as well as a HITS-CLIP data set in activated mouse CD4+ T cells [6]. The local sequence context of the upstream and downstream 30 nt regions flanking the 6-mer seed match are represented using weighted degree kernels [18], which encode position specific *k*-mers for *k* = 1 … 6 (see Materials and Methods). The positions of 3’ ends of alternative 3’UTR isoforms were identified from a human 3’-seq tissue atlas [19] and a mouse PolyA-seq atlas [20]. For each site in human or mouse, positional information was encoded by a vector of distance values (measured in nucleotides) to the annotated stop codon and to the nearest mapped 3’ ends and transformed using a radial basis kernel (see Materials and Methods), and the sum of the weighted degree kernels and positional radial basis kernel was used to train the SVM. In order to model differences in AGO binding preferences between the two data sets—both due to protocol differences and potentially due to cell-type specific factors influencing AGO occupancy—we used multi-task learning to train cell-type specific AGO preference models together with a common AGO binding model (Fig 1B, Materials and Methods). The cell-type specific models are intended to absorb sequence signals that predict AGO binding in a context-dependent manner, while the common model can be used for target prediction in any new context.

### ChimiRic’s duplex model outperforms existing methods for predicting miRNA-mRNA interactions supported by chimeric reads

**Fig 2:**
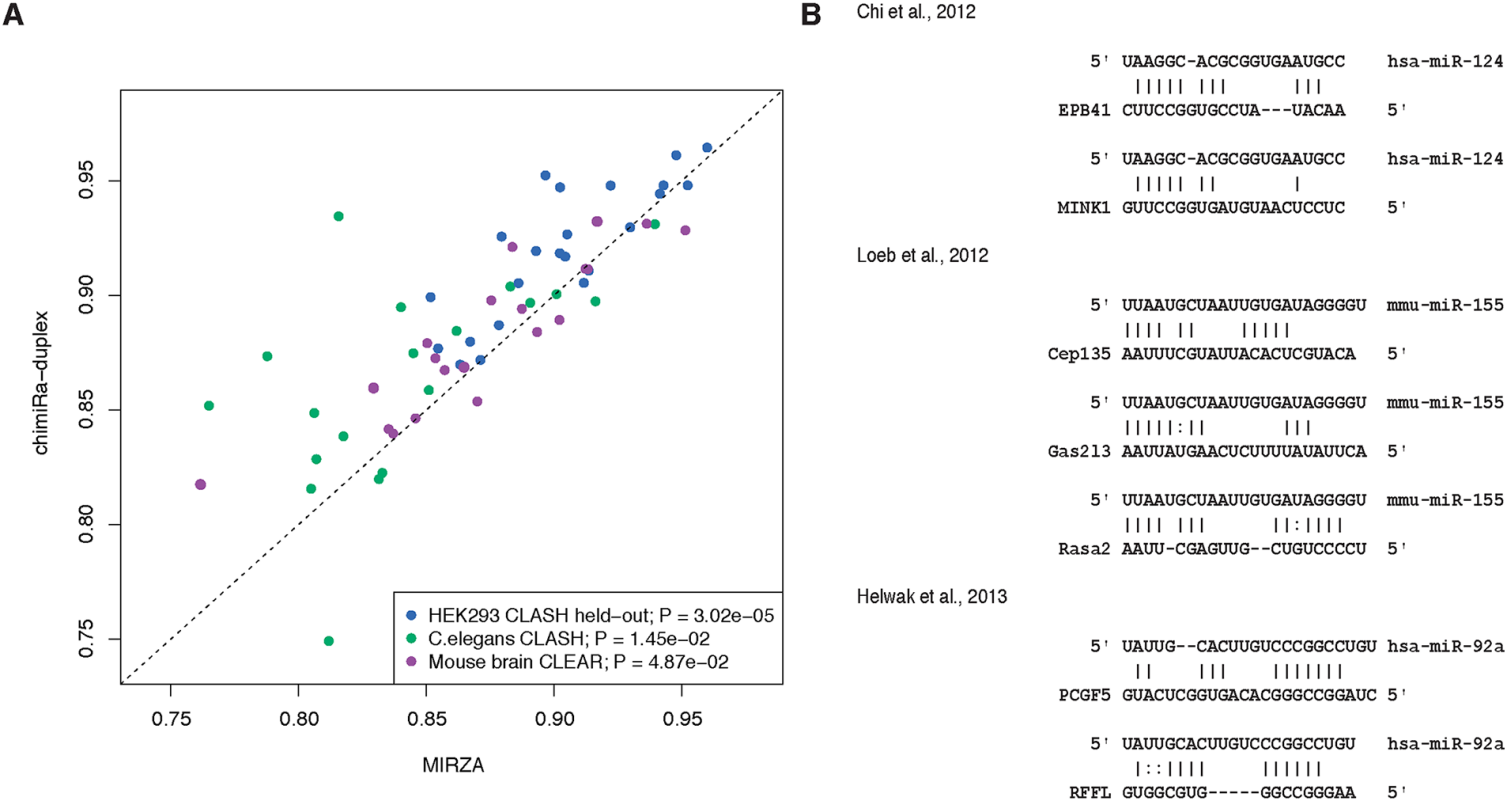
Performance of chimiRic’s duplex model for predicting miRNA-mRNA interactions supported by chimeric reads. (**A**) Duplex model’s performance for predicting the correct interacting miRNA seed family among miRNA-mRNA interactions supported by CLASH chimeric reads. For each miRNA seed family tested, all CLASH-supported interactions for miRNAs in the family are held out from training and form the positive test set; negative test examples consist of interactions for a collection of miRNAs that are held out from training in all experiments. Each point represents the held-out auROC for one of the top 23 miRNA seed families in HEK293 (blue), top 19 miRNA seed families in *C*. *elegans* (green) and top 20 miRNA seed families in mouse brain (purple). (**B**) Examples of duplexes predicted by the model for previously validated non-canonical miRNA-mRNA interactions. Various non-canonical miRNA-mRNA interaction modes were represented, including GU wobbles, bulges and mismatches within seed sequences and interactions relying on 3’ base pairing instead of seed pairing.

To evaluate chimiRic’s duplex model, we held out from training all HEK293 CLASH interactions for a single miRNA seed family (positive test examples) together with a collection of targets sites that interact with other miRNAs based on chimeric read evidence (negative test examples), and we assessed whether the model could rank the held-out miRNA family’s true target sites above these other sites. For each held-out miRNA family in turn, we used chimiRic to generate and score the duplexes between miRNAs in the seed family and mRNA site sequences in the test set. We found that the duplex model could more accurately discriminate true from false interactions compared to MIRZA, an existing method for learning miRNA-mRNA interactions from CLIP data, based on area under the ROC curve (auROC) analysis (Fig 2A, blue points, *p* < 3.02e-5, signed rank test). Note that the original MIRZA model was trained on the same HEK293 PAR-CLIP data set as we used to train the duplex model. To further evaluate the performance on independent data sets, we then used the duplex model trained on HEK293 CLIP and CLASH data to predict miRNA-mRNA interactions supported by chimeric reads from iPAR-CLIP in *C*. *elegans* [21] and CLEAR-CLIP in mouse brain [22]. Again, chimiRic’s duplex model outperformed MIRZA for the task of ranking observed interactions for each miRNA seed family above interactions with targets sites of other miRNAs in both *C*. *elegans* (Fig 2A, green points, *p* < 1.45e-2, signed rank test) and mouse brain (Fig 2A, purple points, *p* < 4.87e-2, signed rank test) data sets. These results suggest that chimiRic’s miRNA-mRNA duplex model can generalize across organisms and protocols for mapping miRNA-mRNA interactions.

Previous differential CLIP and CLASH studies have revealed a broad spectrum of non-canonical miRNA-mRNA interaction modes, including GU wobbles, bulges and mismatches within seed sequences, and interactions relying on 3’ base pairing instead of seed pairing [3, 6, 7]. In order to test whether our duplex model captures some of these known patterns of non-canonical binding, we predicted duplexes for a variety of non-canonical miRNA target sites that have been validated by luciferase assays in previous studies (Fig 2B). Our model have not only correctly identified the correct interacting miRNA above the other highly expressed miRNAs, despite the lack of exact 6-mer seed matches, but also produced duplex structures representative of the previously described interaction modes, including GU wobbles, mismatches and bulges in the seed region, and complementary base pairings in the 3’ region (Fig 2B).

### The full chimiRic model outperforms traditional target prediction for discriminating CLIP-supported miRNA binding sites

Next we combined the duplex model with the AGO binding model, which is trained to discriminate between true AGO bound sites containing 6-mer seeds for highly expressed miRNAs and sites with 6-mer seeds that are not supported by AGO CLIP read evidence, based both on local sequence context and positional bias within 3’UTR isoforms. We used a multi-task strategy to train on AGO-bound versus unbound canonical seed sites for highly expressed miRNAs in two AGO CLIP data sets, HEK293 PAR-CLIP [15] and HITS-CLIP in mouse CD4+ T cells [6]. This procedure learned both task-specific SVM models of AGO binding and a common SVM model. The task-specific SVMs may capture protocol-specific CLIP biases and/or cell-type specific AGO binding preferences. For target prediction in a new context where no CLIP data is available, the common SVM provides a “cell-type agnostic” model of AGO sequence and position preferences.

**Fig 3:**
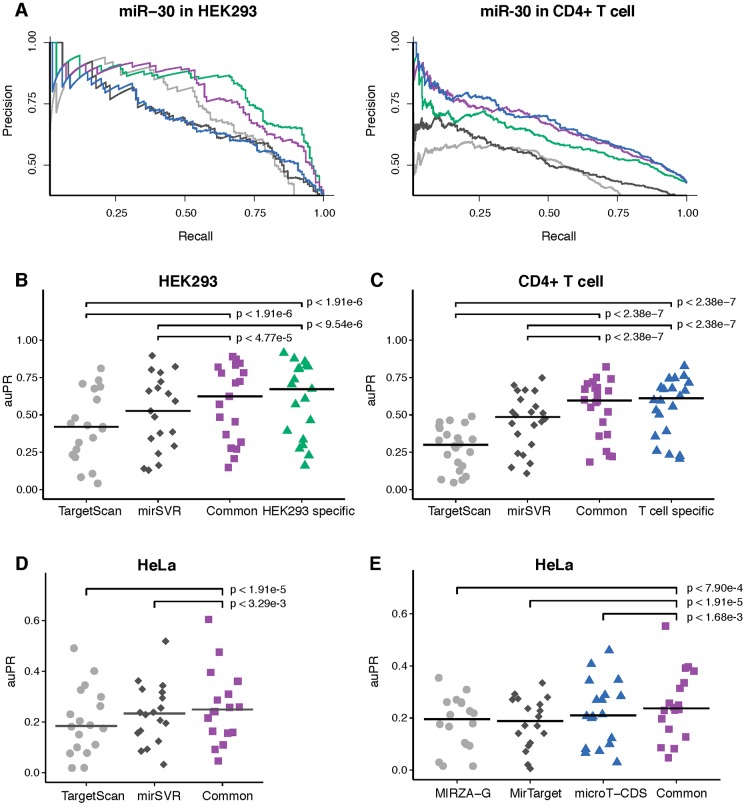
Performance comparison between chimiRic and other methods for discriminating AGO bound sites from unbound sites. (**A**) Examples of precision-recall curves for discriminating AGO-bound canonical target sites from seeds with no AGO support for a single miRNA family (miR-30) in HEK293 and CD4+ T cell. Curves correspond to task-specific (T cell: blue; HEK293: green) and common (purple) AGO binding models, TargetScan (grey) and mirSVR (black). (**B**, **C**) Performance of TargetScan, mirSVR and task-specific/common AGO binding models on held-out miRNA families in HEK293 and CD4+ T cells measured by auPR. Crossbars represent the median auPR of each model. (**D**) Performance of TargetScan, mirSVR and the common AGO binding model on the top miRNA families in an independent HeLa CLIP-seq data set measured by auPR. Crossbars represent the median auPR of each model. (**E**) Performance of MIRZA-G (grey), MirTarget (black), DIANA-microT-CDS (blue) and the common AGO binding model (purple) on the top miRNA families in an independent HeLa CLIP-seq data set measured by auPR. Crossbars represent the median auPR for each model.

To evaluate the combined chimiRic model, for each miRNA seed family, we held out all HEK293 positive target site sequences—both canonical and non-canonical sites supported by chimeric reads from CLASH as well as canonical sites with AGO CLIP read evidence that can be unambiguously assigned to the seed family—and negative site sequences, for training both the duplex and AGO binding models. We then asked how well the combined model performs at discriminating AGO-bound from unbound canonical sites relative to TargetScan [4, 23] and mirSVR [8], two widely used miRNA target prediction algorithms. Fig 3A shows precision-recall curves for the combined chimiRic duplex and HEK293-specific AGO binding model as well as for TargetScan and mirSVR for prediction of canonical sites for several miRNA families. Since TargetScan requires greater seed complementarity than the canonical 6-mer seed (either 7-mer 1A or complementary at miRNA positions 2–8), its overall recall of biochemically-defined sites is limited (note that while the TargetScan 7.0 release discusses 6-mer seeds and non-canonical seeds [23], only a very small fraction of sites were non-canonical in the prediction download files). Evaluating performance by area under the precision-recall curve (auPR) across held-out miRNA seed families showed that this performance advantage was significant over TargetScan (Fig 3B, *p* < 1.91e-6, signed rank test) and mirSVR (Fig 3B, *p* < 9.54e-6, signed rank test). Moreover, even measuring performance up to 50% recall (auPR50), where there are still AGO-bound 7-mer sites to detect, chimiRic still outperformed TargetScan on held-out miRNAs in the HEK293 and T cell data sets (S2 Fig). We then tested the combination of chimiRic’s duplex model and the common AGO binding model. Again we found that chimiRic significantly outperformed TargetScan (Fig 3B, *p* < 1.91e-6, signed rank test) and mirSVR (Fig 3B, *p* < 4.77e-5, signed rank test) on held-out miRNA seed families in HEK293, with minor difference in chimiRic’s performance compared to the HEK293-specific model. Similarly, when predicting the biochemically defined target sites of held-out miRNA families in CD4+ T cells, chimiRic’s duplex model combined with either the T cell specific or the common AGO binding model outperformed TargetScan (Fig 3C, *p* < 2.38e-7 and *p* < 2.38e-7, signed rank tests) and mirSVR (Fig 3C, *p* < 2.38e-7 and *p* < 2.38e-7, signed rank tests). As an independent validation, we also evaluated chimiRic’s performance in a third cellular context using two HITS-CLIP data sets in HeLa cells [1, 7]. Again, we found that the common AGO binding model combined with duplex model had a significant advantage over TargetScan (Fig 3D, *p* < 1.91e-5, signed rank test) and mirSVR (Fig 3D, *p* < 3.29e-3, signed rank test). Evaluation using auPR50, which favors TargetScan by allowing reduced recall, still showed a significant performance advantage of the common chimiRic model over TargetScan and mirSVR in HEK293 and T cells, with a statistical tie on the HeLa cells (S2 Fig). We also evaluated the performance of three additional methods, MIRZA-G [24], MirTarget [13] and DIANA-microT-CDS [10], all of which are trained on AGO CLIP data and provide one a single prediction score for each miRNA-gene interaction. When we compared the performance on the same HeLa data set, the common chimiRic model outperformed all three methods measured by auPR (Fig 3E, *p* < 7.90e-4, *p* < 1.91e-5 and *p* < 1.68e-3, signed rank test), partly due to chimiRic’s better recall. When measured by auPR50, chimiRic still achieved a statistical tie against these methods (S2 Fig), showing that chimiRic’s top-ranked predictions are at least as accurate as other methods trained on AGO CLIP data sets.

We also tested our performance relative to a typical evaluation of miRNA target prediction methods: predicting the extent of mRNA downregulation of miRNA targets. We evaluated the performance of TargetScan, mirSVR and chimiRic on eight miRNA transfection experiments in HCT116 cells [25]. Despite the fact that chimiRic was not trained on any expression data, the top predictions of chimiRic conferred a similar amount of regulation compared to TargetScan, while achieving better performance than mirSVR (S3 Fig).

### AGO-binding model learns 3’UTR positional preferences and RNA-binding motifs associated with miRNA targeting

**Fig 4:**
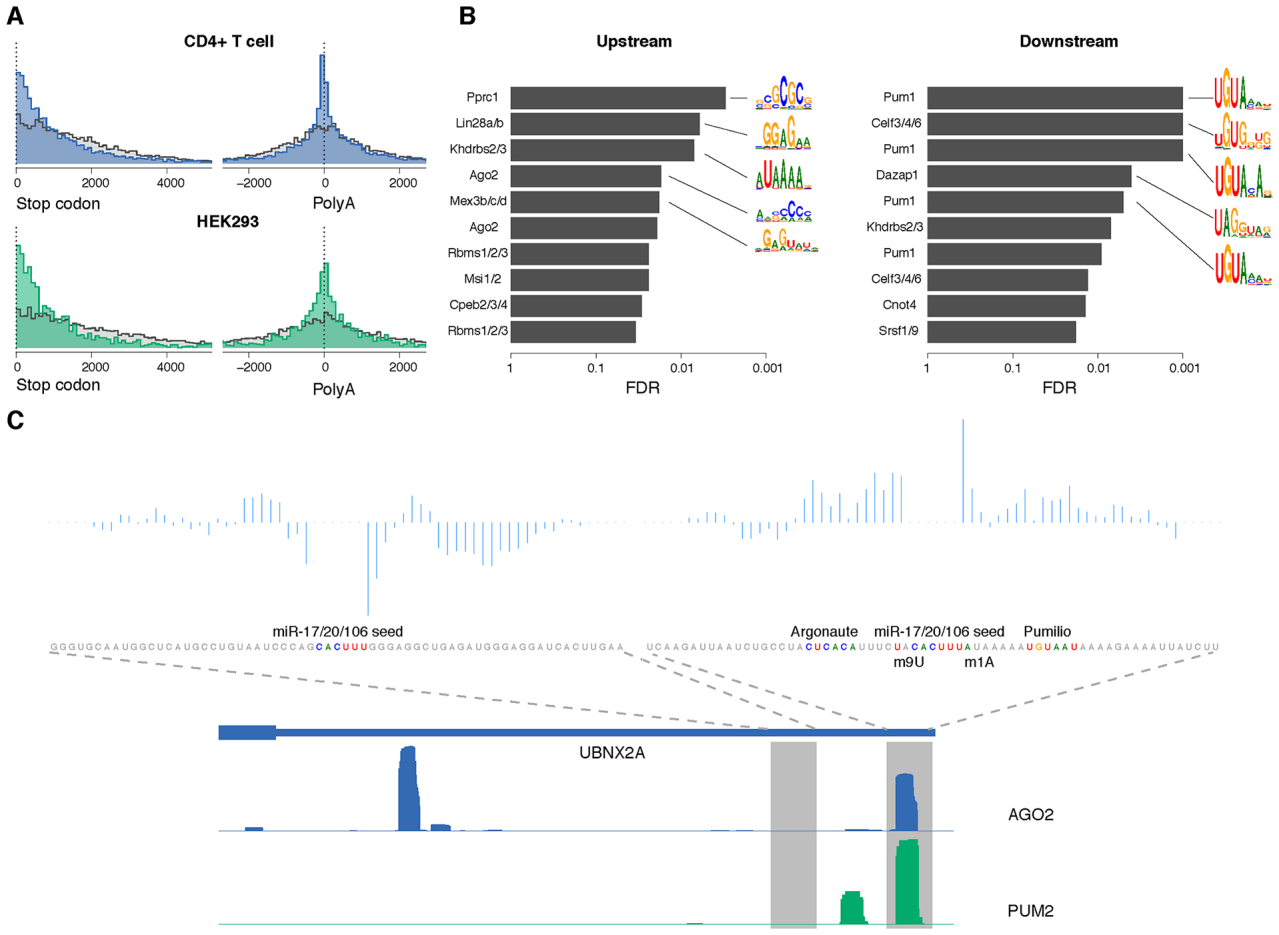
Interpretation of the AGO-binding model learned from CLIP-seq data. (**A**) Positional distribution of AGO binding sites (blue/green) and unbound sites (grey) within 3’UTRs in CD4+ T cell (top) and HEK293 (bottom), showing enrichment of bound sites near the start of the 3’UTR (left) and in the region upstream of internal 3’ cleavage sites of multi-UTR transcripts (right). There is also enrichment of AGO-bound sites ~200nt downstream of internal 3’ cleavage sites, suggesting that the resolution of the PolyA-seq peaks can be limited and/or that clusters of nearby 3’ cleavage sites confound the analysis. All distances were between the position aligned against nucleotide 2 of the miRNA and the start/end of the corresponding 3’UTR. (**B**) RBPs with motifs that match the most discriminative *k*-mers in the common sequence model. Positions with the highest differential POIM for 6-mers upstream and downstream of the miRNA seeds were chosen, and then a signed rank test was used to assess the enrichment of POIM *k*-mers in RNAcompete array probes. False discovery rates (FDRs) were estimated using the empirical *p*-value distribution from 1,000 SVMs trained on random permutations of the +/- labels. Motif logos summarized from the original RNAcompete assays are shown for the top 5 RBPs as ranked by FDR. (The same RBP symbol may appear multiple times since in some cases several constructs of the same protein were assayed by RNAcompete.) (**C**) An example of co-binding of Pumilio and Argonaute at miRNA target sites. Two miR-17/20/106 seed matches within the 3’UTR of UBNX2A are shown, one with AGO2 binding and one without, along with the coverage profiles of AGO2 and PUM2 CLIP in HEK293. For each site, the prediction scores from the SVM sequence model are decomposed into positional scores and displayed. Sequence features near the target site including the Argonaute motif, Pumilio motif, m1A and m9U are also highlighted.

Previous studies have suggested that 3’UTR miRNA target sites tend to reside near the stop codons or near the 3’ end of the transcript rather than the middle of 3’UTRs [4]. We confirmed a positional enrichment of AGO-bound sites near the stop codons (Fig 4A, top) and near the end of the 3’UTR compared to miRNA seeds with no AGO binding in CD4+ T cells across mouse transcripts. Additionally, for multi-UTR transcripts, we observed an enrichment of AGO-bound sites in the region upstream of internal 3’ cleavage sites (as mapped by PolyA-seq) that was absent for the negative site examples (Fig 4A, top, *p* < 2.2e-16, KS test). We also observed an enrichment of positive site examples ~200nt *downstream* of internal cleavage sites, suggesting that the resolution of the mapped 3’ ends in the mouse atlas is limited and/or that clusters of nearby 3’ cleavage sites confound the analysis. Likewise, we found HEK293 AGO binding sites enriched upstream of internal 3’ cleavage sites based on the human 3’ end atlas (mapped by 3’-seq), with more modest downstream enrichment (Fig 4A, bottom). These positional biases are encoded in the feature representation for the AGO binding model (see Materials and Methods) and lead to a significant performance improvement for the full chimiRic model (mean auROC on held-out miRNA families of 0.775 without positional bias information vs. 0.849 in the full model, *p* < 2.38e-7, signed rank test; S4 Fig).

To further interpret the sequence features in the AGO binding model, we used the positional oligomer importance matrix (POIM) [26] approach to identify the significant positional *k*-mers. From the 1-mer POIMs, we observed not only high AU content flanking the miRNA seed matches in general but also specific positional signals like m1A and m8/9U (S5 Fig), which are consistent with findings from previous studies [5, 17]. Moreover, the representation allowed us to go beyond single nucleotide composition, which is the extent of sequence contextual information used in most previous miRNA target prediction methods, to explore more complex sequence features.

Previous studies have suggested that various RNA binding proteins (RBPs) can bind to regions proximal to miRNA target sites in order to enhance or repress miRNA-mediated regulation [27–29]. Therefore, one potential explanation for the long positional *k*-mers that discriminate between AGO binding sequences and unbound sequences is that they correspond to the motifs of co-binding RBPs that mediate AGO occupancy. To explore this hypothesis, we matched the 6-mers from positions with top differential POIM scores to RNAcompete *in vitro* affinity data for a compendium of RBPs [30, 31]. By measuring the enrichment of these *k*-mers in RNAcompete data across all RBPs and assessing significance relative to an empirical null model based on training SVMs on random permutations of the class labels (see Materials and Methods), we found that the position-specific *k*-mers in upstream and downstream sequences were indeed consistent with several known RBP motifs (Fig 4B). In the common AGO-binding model, we identified an AC-rich motif upstream of the seed match that matched an AGO RNAcompete experiment and has been proposed to be the miRNA-independent binding signal for Argonaute [31]. Meanwhile, in the downstream component of the common model, Pumilio was identified as the most significant RBP motif. It has been previously suggested that Pumilio has a role in regulating miRNA site accessibility of specific target genes [28, 32, 33]. Our analysis suggests that Pumilio may play a transcriptome-wide role in mediating AGO binding. We compared the HEK293 AGO CLIP to PUM2 PAR-CLIP in the same cell type [2] and found that 16.4% of AGO sites in HEK293 overlapped with PUM2 binding sites. Fig 4C shows one example of a miR-17/20/106 target site in the 3’UTR of UBNX2A together with sequence signals identified by the model. After decomposing the SVM sequence scores into positional prediction scores (see Materials and Methods), we found that the positions with positive contribution overlapped exactly with the Pumilio binding motif and Pumilio CLIP coverage. In contrast, another miR-17/20/106 seed match site in the same 3’UTR was not bound by AGO and lacked significant positional *k*-mers from the sequence model.

## Discussion

We have presented an integrative model for predicting miRNA binding sites by training on sequencing assays that map biochemical interactions via AGO cross-linking and miRNA-mRNA ligation. We demonstrated that chimiRic can detect non-canonical miRNA-mRNA binding modes and significantly outperforms MIRZA for predicting the interacting miRNA for both canonical and non-canonical mRNA target sites. Moreover, chimiRic outperforms TargetScan, a leading target prediction method, for discriminating canonical seed sites that are bound by AGO from unbound sites. The feature representation of our AGO binding model exploits recent 3’-end sequencing data that identifies alternative 3’UTR isoforms and enables analysis of mRNA sequence signals in the vicinity of the miRNA binding sites, suggesting that other RBPs may collaborate with AGO to mediate miRNA-mRNA interactions.

ChimiRic directly predicts miRNA targeting by learning from miRNA binding data, whereas most existing algorithms infer miRNA targets and model their efficiency using mRNA expression changes in miRNA overexpression experiments in cell culture [4, 8]. One major issue with methods trained solely on gene expression changes is that the direct effects of miRNA regulation are confounded with secondary effects, leading to label noise in the learning problem. Since the true binding sites that mediate direct regulation are unknown in this setting, inference of miRNA targets involves “bootstrapping” from an initial set of assumptions of what constitutes a viable target. Furthermore, miRNA transfections in cell culture represent a non-physiological context for miRNA activity and may not accurately reflect endogenous targeting rules. Finally, miRNA binding can inhibit translational efficiency of target mRNAs in addition to or instead of reducing mRNA abundance [34]. While previous global studies suggest that miRNA-mediated changes at the mRNA and protein levels are correlated, these data also depend on miRNA overexpression in cell lines [35, 36]. For all these reasons, it is possible that what we have already exhausted what can be learned indirectly from mRNA expression changes due to miRNA perturbations—and from miRNA overexpression experiments in particular—and that new AGO CLIP and CLASH technologies for mapping direct interactions are required to advance our understanding of miRNA targeting in cells.

However, recent assays for mapping AGO sites and miRNA-mRNA interactions are technically difficult and present significant challenges for computational analysis and training of predictive models. CLASH and similar protocols that use RNA ligation to capture miRNA-mRNA interactions currently have very low ligation efficiency (only ~2% of reads are chimeric) [3, 21], suggesting that a large number of miRNA-mRNA interactions remain uncaptured. Some non-canonical interactions recovered by CLASH may be due to artifacts or biases in the ligation experiments, and one previous study found that incorporating chimeric reads into MIRZA did not significantly improve prediction performance [24]. Even in the more mature CLIP assays, data reproducibility is still limited and strongly affected by technical differences between various protocols (e.g. PAR-CLIP, HITS-CLIP, iCLIP) that produce protocol-specific biases [15] and by the potential false positives resulted from background binding [37]. In our experiments, we only trained on data sets with multiple biological replicates in order to ensure saturating coverage and to correctly label the mRNA sites as positive or negative. We further used a multi-task strategy to absorb dataset-specific differences into task-specific models and learn a common model that captures general sequence signals and positional preferences of AGO binding. Although the extent of miRNA target context-specificity remains unclear [38, 39], it is still possible that there are true biological differences in AGO occupancy between cell types. Indeed, even directed perturbation of a single miRNA-mRNA interaction can lead to distinct changes in functional responses in different immune cell types [40]. Ultimately, as CLIP-based technologies mature and larger data sets accrue, the algorithmic approaches we present here may reveal the RNA sequence elements and trans-acting factors that mediate cell-type specific miRNA-mRNA interactions.

## Materials and Methods

### Processing of the sequencing data

Argonaute PAR-CLIP data in HEK293 cells [15], HITS-CLIP in mouse CD4+ T cells [6] and HITS-CLIP in HeLa cells [1, 7] were used to define the miRNA target sites in mRNA sequences. Reads from the wild-type libraries were aligned to hg19 and mm9 genome using the bwa aligner [41]. Argonaute binding sites were then identified from the coverage profile of uniquely aligned reads using a previously described peak calling algorithm [19]. Chimeric reads from CLASH in HEK293 cells [3], iPAR-CLIP in *C*. *elegans* [21], and CLEAR-CLIP in mouse brain [22] were also used for training (HEK293) and testing (*C*. *elegans* and mouse brain) the duplex model. We used the list of miRNA-mRNA interactions provided in the original publications with additional filtering. Interactions were chosen according to following criteria: (1) binding sites were located in the 3’UTR; (2) binding sites contained complementary matches to the interacting miRNA 6-mer seed with edit distance of 0 or 1; (3) interactions were also supported by non-chimeric reads.

3’-seq data in human tissues [19] and PolyA-seq data in mouse tissues [20] were used to construct 3’UTR isoform atlases in human and mouse. The processing procedure was the same as previously described [19].

### Processing of the mRNA expression data

The array data set included gene expression changes in eight individual miRNA transfection experiments in HCT116 cells (miR-15a, miR-16, miR-215, miR-17, miR-20a, let-7c, miR-106b and miR-103a, corresponding to GEO data sets GSM156545, GSM156546, GSM156548, GSM156553, GSM156554, GSM156557, GSM156576 and GSM156580) [25]. To reduce the noise from genes with baseline expressions, we restricted our analysis to probes with signal intensities above median in the control experiments. We also only included the genes with a single potential target site of the transfected miRNA in order to simplify the analysis. The extent of downregulation was represented by the log2 fold change between 24 h post-transfection and control, while genes with multiple probes were represented by the median of all probes.

### Composition of training and testing sets

For the duplex model, each example was a pair of miRNA and mRNA site sequences. If the same interaction was identified by chimeric reads in the CLASH data, then we considered the interaction to be positive. Otherwise, if the site was not interacting with the miRNA or another miRNA from the same seed family, then we considered it as a negative example. Due to the limited number of interactions identified by CLASH, we also added interactions inferred from CLIP data by assuming that Argonaute binding sites containing 6-mer seed matches interacted with the corresponding miRNAs, while sites without Argonaute binding were unlikely to interact with miRNAs. These assumptions provided another set of positive and negative examples of miRNA-mRNA interactions.

For the AGO binding model, each example was a 3’UTR site matched to the 6-mer seed of one of the highly expressed miRNAs in the corresponding cell type (HEK293: 59 miRNAs from 21 miRNA families; CD4+ T cells: 58 miRNAs from 24 miRNA families). If a seed match overlapped with an Argonaute binding site in the CLIP data, we identified it as a positive example for the corresponding miRNA. Otherwise, if a seed overlapped with no Argonaute CLIP reads, we considered it to be a negative example.

### Feature representation for duplex and context models

We adapted the feature representation from MIRZA [12] to describe the duplex structures formed between interacting (miRNA, site) pairs. Three types of features were included in the representation: (1) the type of base pair (GU, UG, AU, UA, GC, CG) at each position in the alignment; (2) the bases where a loop is opened, symmetrically extended or asymmetrically extended in the duplex structure; (3) binary variables for each position in the miRNA sequence representing whether it is paired to an mRNA base or not. One major change we made to the original representation was that the only permissible base pairing of the first base in the miRNA was with an A in mRNA sequence, so that only an A across from position 1 would contribute positively to the score. This restriction is derived from the observations in previous studies [4].

We described the mRNA sites with two types of UTR features: local sequence context and global positional context. The sequence context was represented by positional *k*-mer features (*k* = 1, …, 6) from 30 nt sequences upstream and downstream of the miRNA seed match and implemented using two weighted degree string kernels [18]. Three positional context features for each site were computed as (i) the distance to the nearest stop codon, (ii) the distance to the next end of a 3’UTR isoform, and (iii) the distance to the previous end of a 3’UTR isoform and were renormalized with a radial basis kernel. These local sequence kernel and positional kernel were then combined by summing kernel matrices.

### Training and testing of duplex and context models

We trained the duplex model both on (miRNA, site) examples directly derived from CLASH interactions and on examples with interactions inferred from CLIP based on 6-mer seed complementarity. One major advantage of the miRNA-mRNA duplex representation described above is that the model weights ***w*** can also be used as the parameters for local pairwise alignment [12]: given the feature description *φ*(miRNA, site) for a duplex alignment, the alignment score can be described by the additive scoring function ***w*·***φ*(miRNA, site). Therefore, by iteratively optimizing the model weights given the currents alignments and then computing the optimal alignments given current model weights, we can simultaneously optimize the duplexes and the scoring model. The initial duplex structure for each (miRNA, site) pair was predicted by *duplexfold* in the ViennaRNA package [42], and the corresponding duplex feature vectors were then used to train a linear support vector machine (SVM) classifier. The model weights ***w*** were then used as local alignment parameters to update the duplex structure between the miRNA and mRNA site sequences. The same process was repeated for 12 iterations, by which point the model vector had converged, and the final duplex structures and model weights were used as the duplex model’s output. To compensate for the class imbalance, in each iteration we only used a fraction of negative examples randomly sampled from the whole set while using all positive examples. Specifically, we sampled 15 times as many CLASH negatives as CLASH positives, and the same number of CLIP negatives as CLIP positives.

We applied a regular SVM classifier to the UTR kernel matrix when we trained the AGO binding model using CLIP training data from a single cell type. When we combined data sets from multiple cell types, we applied the multi-task learning approach [43] and treated the different cell types as different but related learning tasks to address the possibility of cell type specific miRNA targeting and AGO binding rules as well as protocol specific biases. We implemented the multi-task SVM as a modification to the kernel matrix:

$$K_{st}\left(x,z\right)=\left(\mu +\delta_{st}\right)\;K\left(x,z\right)$$

If two examples x and z belong to the same task (in other words, two sites were from the same cell type), then an extra weight is added to their product in the kernel matrix to reflect the relationship. The free parameter μ controls the closeness of task-specific models to the average model, and its optimal value was determined by five-fold cross-validation.

All the machine learning procedures described above were implemented with Numpy (http://www.numpy.org) and the Shogun machine learning tool box (http://www.shogun-toolbox.org).

### Method comparison

The latest TargetScan 7.0 predictions for human and mouse (context++ scores) were downloaded from http://www.targetscan.org and mirSVR predictions for human and mouse were downloaded from http://www.microrna.org. For both methods, if any target site had multiple possible interacting miRNAs, we used the interaction with the highest prediction score. Predictions for human genes from MIRZA-G (seed-MIRZA-G-C variant), DIANA-microT-CDS and MirTarget were downloaded from http://www.clipz.unibas.ch/index.php?r=tools/sub/mirza_g, http://diana.imis.athena-innovation.gr/DianaTools/index.php?r=microT_CDS/index and http://mirdb.org. Since these methods provide one single prediction score for each miRNA-gene interaction, for this comparison we also simplified our predictions by using the highest score for genes with multiple sites for the same miRNA.

### Search for potential RNA binding protein motifs near miRNA target sites

In order to interpret the local sequence context features captured by the learned AGO binding SVM, we computed the positional oligomer importance matrices (POIMs) [26] for the upstream and downstream weighted degree string kernels, which represent the positional *k*-mer features enriched in Argonaute binding sequences. For both POIMs, we chose the positional 5-mer or 6-mer with the highest differential POIM weight and used 15 *k*-mers with highest POIM weights from that position to represent the most significant motifs within the positive sequences. We then matched them to potential RNA binding protein motifs identified by RNAcompete assays [30, 31]. Normalized array probe intensities for 208 RBPs were downloaded from the supplemental websites (http://cisbp-rna.ccbr.utoronto.ca; http://www.cs.toronto.edu/~taehyung/gr_ago.html). For each RNAcompete experiment, we selected the top 1000 probe sequences with highest intensity and performed a one-sided Wilcoxon rank-sum test comparing the probes with and without the top *k*-mers to test the significance of enrichment.

Due to the biased nucleotide content near miRNA targets, it is necessary to estimate false discovery rates (FDRs) for the statistical tests. For each RNAcompete experiment, we generated an empirical null distribution of *p*-values by training the AGO-binding SVM models 1,000 times with randomly permutated labels, extracting the top POIM *k*-mers with the same *k* and position as in the real model, and testing the enrichment of the top *k*-mers from the random models within the top probes. The FDRs were then computed by converting the enrichment *p*-values from the real model to empirical *p*-values from the 1,000 rounds of permutations. To better relate enriched *k*-mer signals to the biological context, we also filtered out RBPs with no homologs in mouse and human according to cisBP-RNA (http://cisbp-rna.ccbr.utoronto.ca) or with low mRNA abundance according to RNA-seq data in the same cell types [3, 6]. Of the remaining RBPs, we considered the top 5 as ranked by FDR as the ones with potential motif enrichment near the miRNA target sites.

To examine the contribution of positional *k*-mer features at specific binding sites, we decomposed the SVM score by summing up the SVM weights for all *k-*mers from the same position in the upstream/downstream sequence model. One example was visualized in Fig 4C to show the overlap between RBP binding and the corresponding sequence signals.

## Supporting Information

S1 Fig Illustration of the iterative learning process of the duplex model. An advantage of the miRNA-mRNA duplex representation is that the model weights ***w*** can be used as the parameters for local pairwise alignment: given the feature description *φ*(miRNA, site) for a duplex alignment, the alignment score can be described by the additive scoring function ***w*·***φ*(miRNA, site). The initial duplex structure for each (miRNA, site) pair was predicted by *duplexfold* in the ViennaRNA package, and the corresponding duplex feature vectors were used to train a linear support vector machine (SVM) classifier. The model weights ***w*** were then used as local alignment parameters to update the duplex structure between the miRNA and mRNA site sequences. The same iterative process was repeated until convergence of the duplex model.
(PDF)

S2 Fig Performance comparison between chimiRic and other methods for discriminating AGO bound sites from unbound sites. Area under the precision-recall curve up to 50% recall (auPR50) was used as an alternative metric to compensate for the fact that TargetScan in general has lower recall due to omission of 6-mer seed match sites. (**A**, **B**) Performance of TargetScan, mirSVR and task-specific/common chimiRic AGO binding models on held-out miRNA families in HEK293 and CD4+ T cells measured by auPR50. Crossbars represent the median auPR50 of each model. (**C**) Performance of TargetScan, mirSVR and the common chimiRic AGO binding model on the top miRNA families in an independent HeLa CLIP-seq data set measured by auPR50. Crossbars represent the median auPR50 of each model. (**D**) Performance of MIRZA-G, MirTarget, DIANA-microT-CDS and the common chimiRic AGO binding model on the top miRNA families in an independent HeLa CLIP-seq data set measured by auPR50. Crossbars represent the median auPR50 of each model.
(PDF)

S3 Fig Performance comparison of chimiRic, TargetScan and mirSVR for predicting target downregulation in miRNA transfection assays. The extent of mRNA downregulation for the top N predictions of each method (chimiRic: purple; TargetScan: grey; mirSVR: black), including **(A)** or excluding **(B)** predicted 6-mer seed sites. The extent of downregulation was represented by the median log2 fold changes between transfection and control, while the variation between eight data sets was represented by standard error bars.
(PDF)

S4 Fig Contribution of positional features to the performance of AGO binding model. Each data point represents the performance on one held-out miRNA family in HEK293 CLIP data set, where the x-axis represents the auROC of the chimiRic AGO binding model without positional features and the y-axis represents the auROC of the full chimiRic AGO binding model.
(PDF)

S5 Fig Interpretation of single-nucleotide features in the common AGO binding model via 1-mer POIMs. The POIMs for upstream/downstream positional 1-mer components of the common chimiRic AGO binding model are visualized as heatmaps. Position 1 in downstream and upstream sequences matches nucleotide 1 and nucleotide 8 in the miRNA, respectively. Therefore the most significant single-nucleotide features correspond to m1A and m8/9U.
(PDF)

S1 Table Top miRNA families used in CLIP-seq training and testing data sets. (XLSX)
