# Supplementary material for: Learning to Predict miRNA-mRNA Interactions from AGO CLIP Sequencing and CLASH Data
Source: PLoS Comput Biol. 2016 Jul 20;12(7):e1005026. doi: 10.1371/journal.pcbi.1005026 (PMC4954643; doi:10.1371/journal.pcbi.1005026)
Supplement: S1 Fig — An advantage of the miRNA-mRNA duplex representation is that the model weights w can be used as the parameters for local pairwise alignment: given the feature description φ(miRNA, site) for a duplex alignment, the alignment score can be described by the additive scoring function w·φ(miRNA, site). The initial duplex structure for each (miRNA, site) pair was predicted by duplexfold in the ViennaRNA package, and the corresponding duplex feature vectors were used to train a linear support vector machine (SVM) classifier. The model weights w were then used as local alignment parameters to update the duplex structure between the miRNA and mRNA site sequences. The same iterative process was repeated until convergence of the duplex model. (PDF) [file pcbi.1005026.s001.pdf]

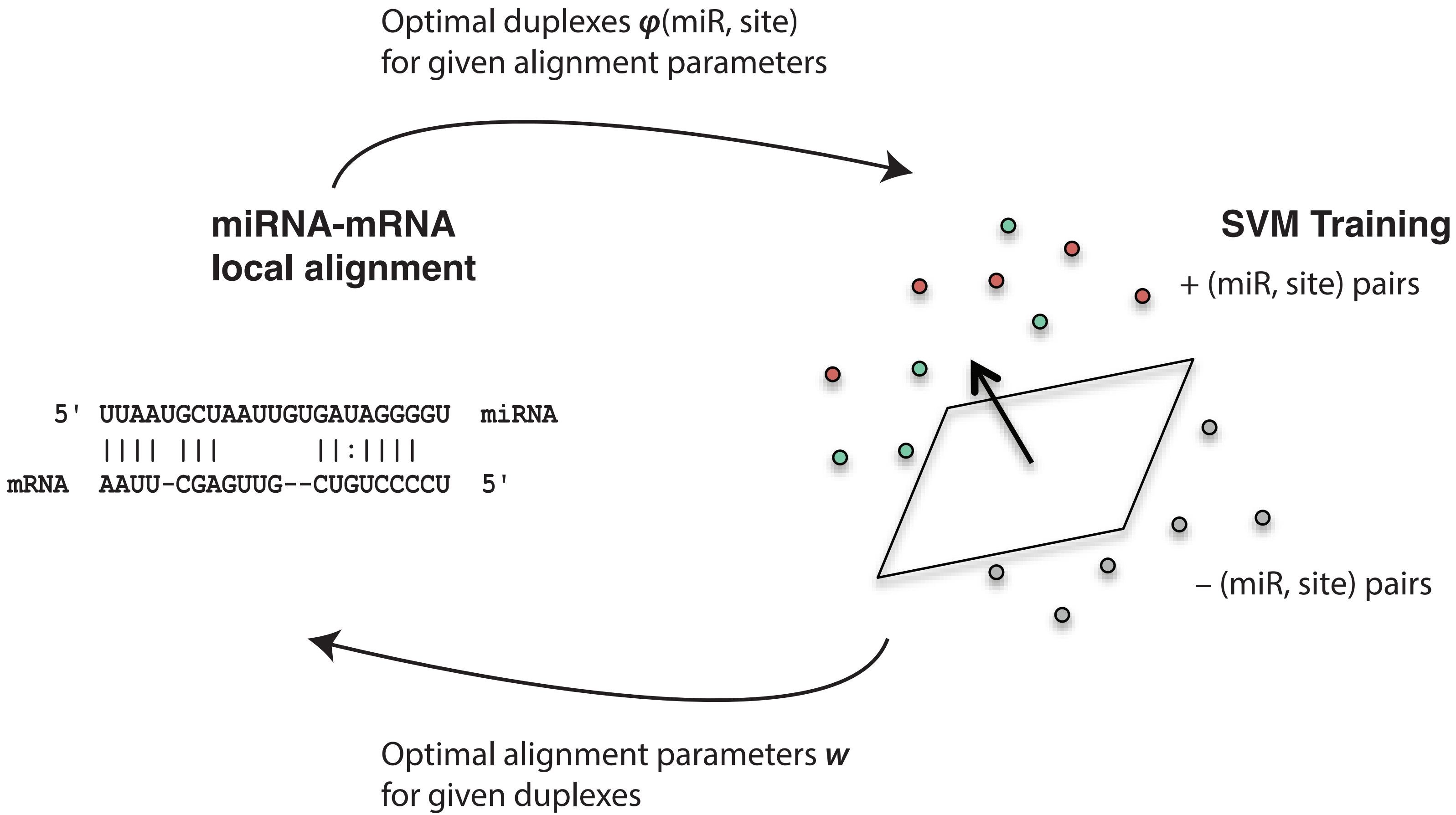
Optimal duplexes φ(miR, site)
for given alignment parameters
miRNA-mRNA
local alignment
5' UUAAUGCUAAUUGUGAUAGGGU miRNA
|||| ||| ||:||||
mRNA AAUU-CGAGUUG--CUGUCCCCU 5'
SVM Training
+ (miR, site) pairs
– (miR, site) pairs
Optimal alignment parameters w
for given duplexes
