# Supplementary figures and images for: Learning to Predict miRNA-mRNA Interactions from AGO CLIP Sequencing and CLASH Data
Source: PLoS Comput Biol. 2016 Jul 20;12(7):e1005026. doi: 10.1371/journal.pcbi.1005026 (PMC4954643; doi:10.1371/journal.pcbi.1005026)

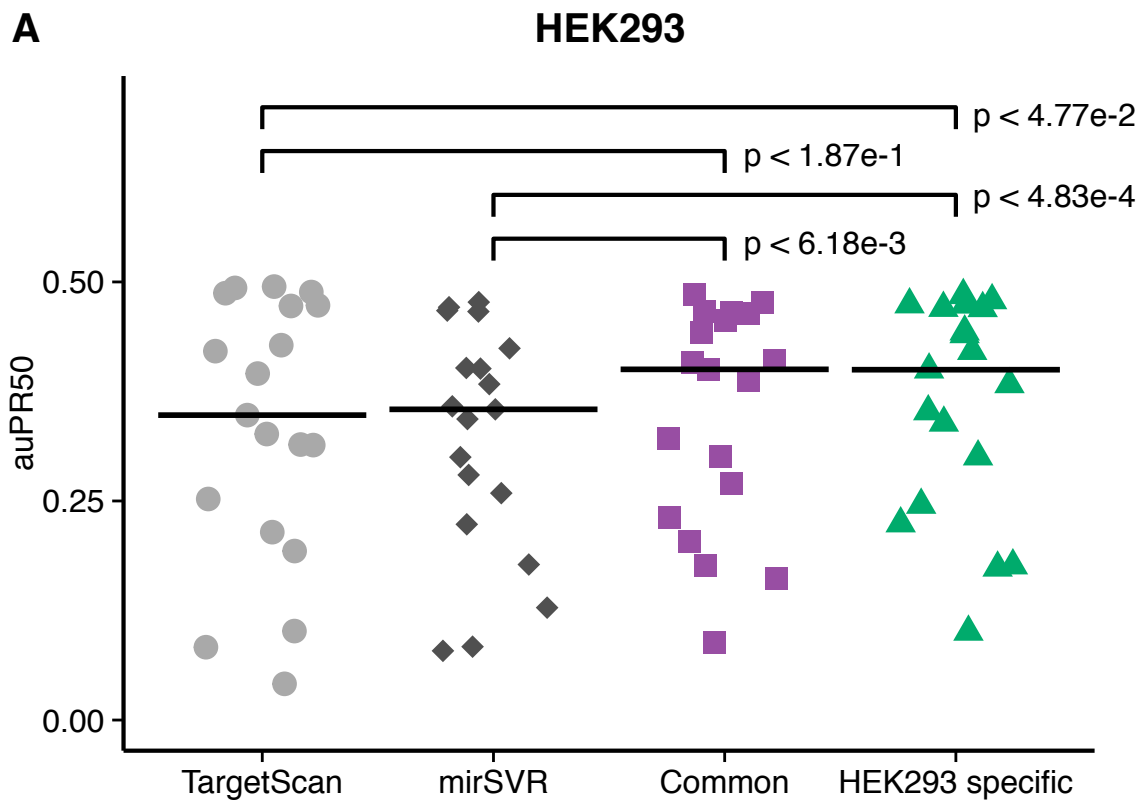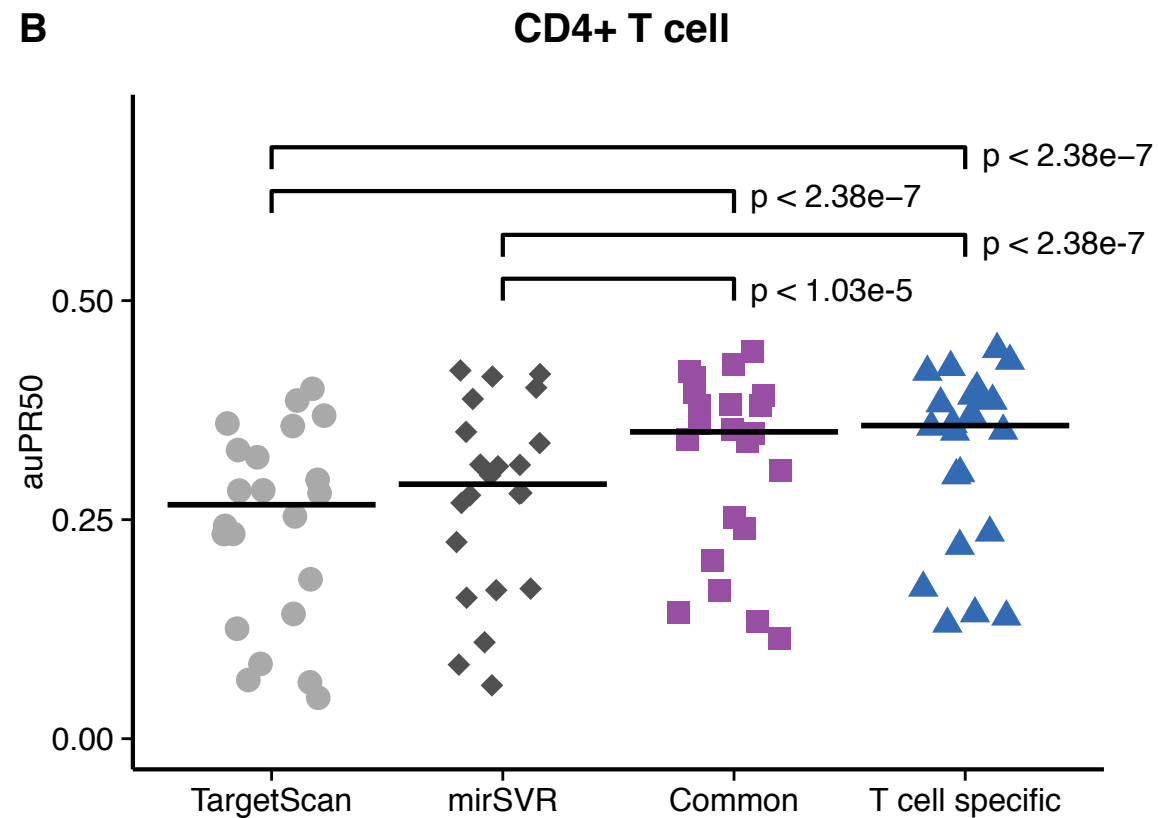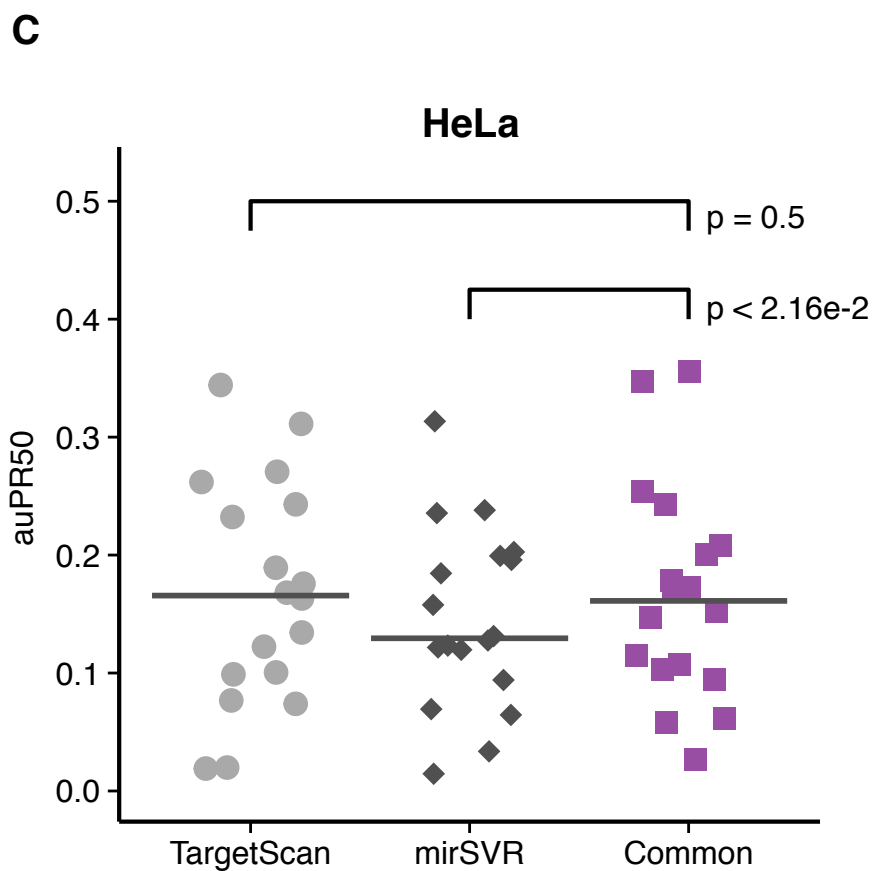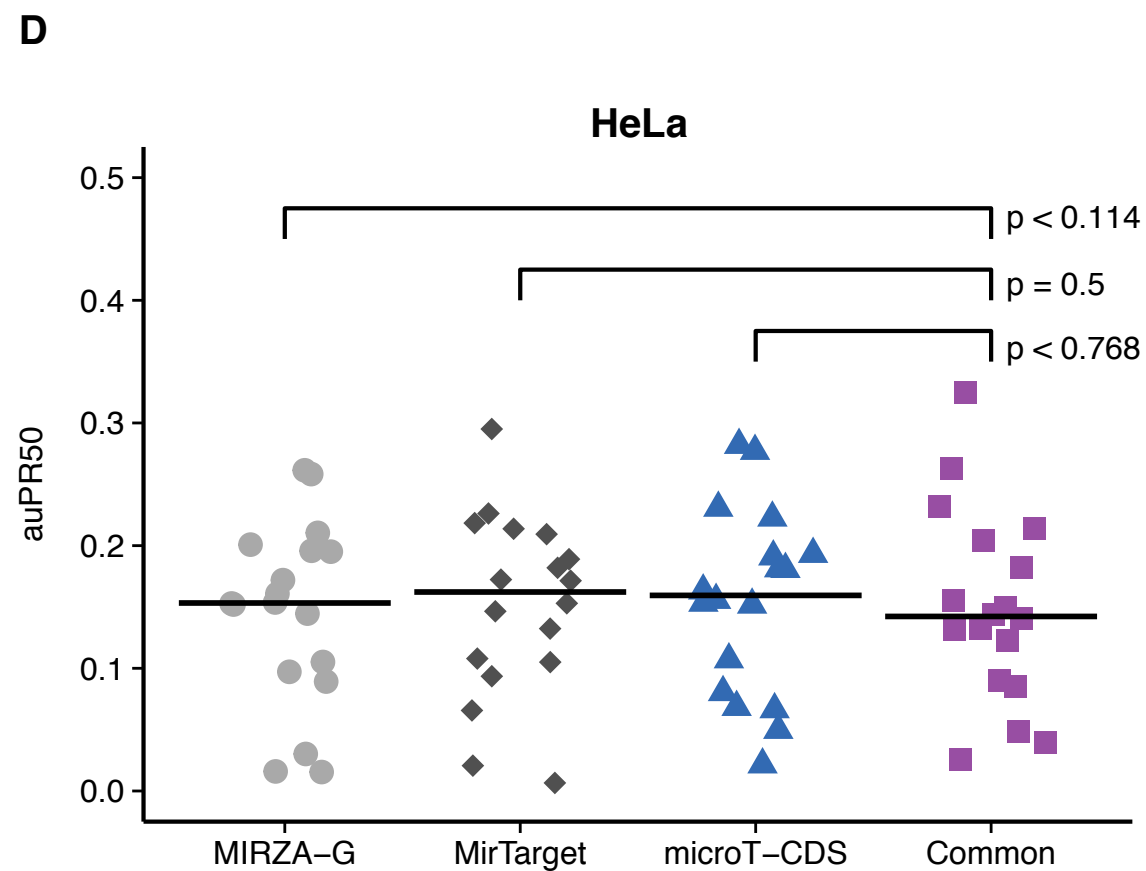

Supplement: S2 Fig — Area under the precision-recall curve up to 50% recall (auPR50) was used as an alternative metric to compensate for the fact that TargetScan in general has lower recall due to omission of 6-mer seed match sites. (A, B) Performance of TargetScan, mirSVR and task-specific/common chimiRic AGO binding models on held-out miRNA families in HEK293 and CD4+ T cells measured by auPR50. Crossbars represent the median auPR50 of each model. (C) Performance of TargetScan, mirSVR and the common chimiRic AGO binding model on the top miRNA families in an independent HeLa CLIP-seq data set measured by auPR50. Crossbars represent the median auPR50 of each model. (D) Performance of MIRZA-G, MirTarget, DIANA-microT-CDS and the common chimiRic AGO binding model on the top miRNA families in an independent HeLa CLIP-seq data set measured by auPR50. Crossbars represent the median auPR50 of each model. (PDF) [file pcbi.1005026.s002.pdf]

**A****6/7/8-mers**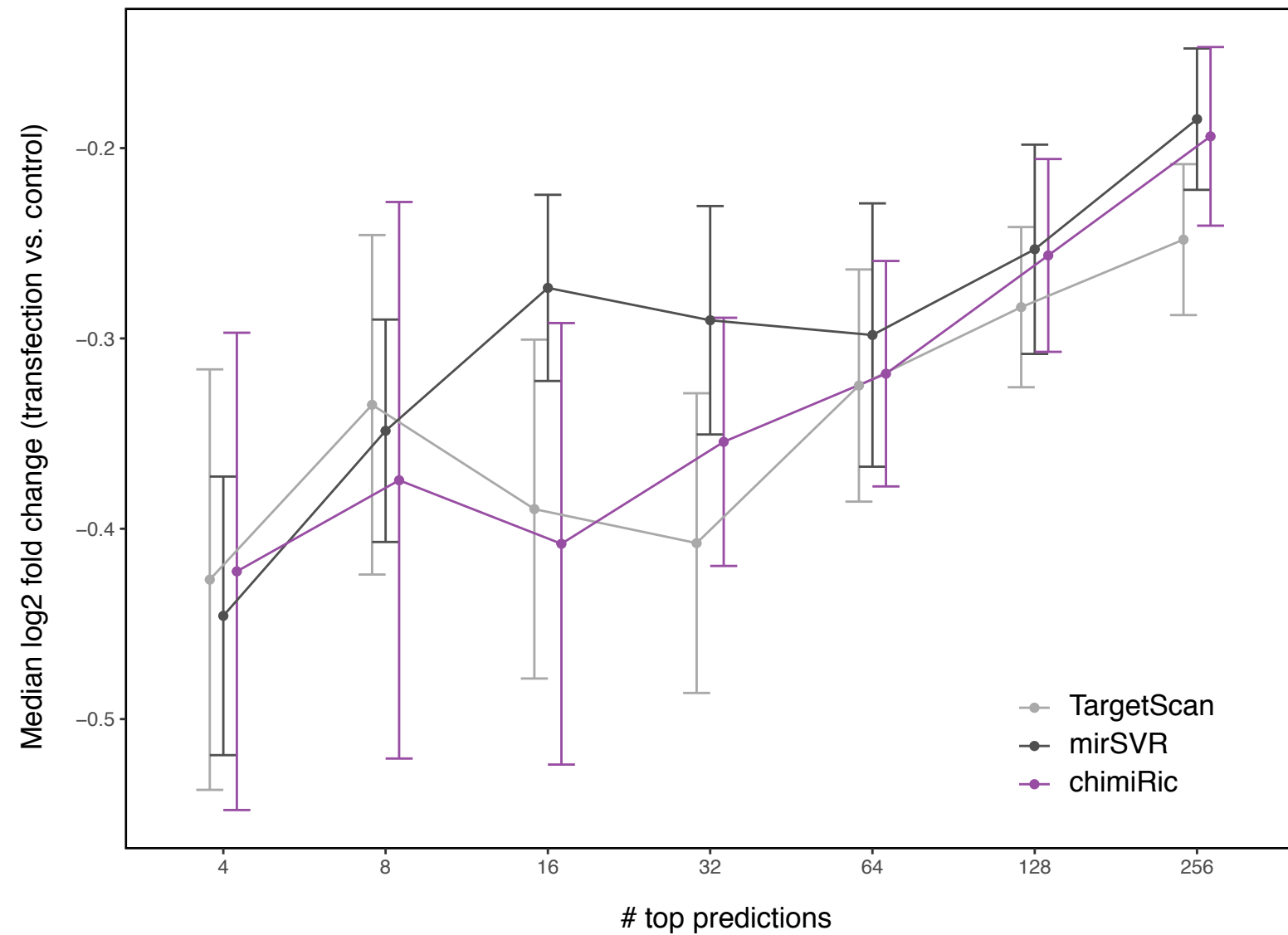**B****7/8-mers**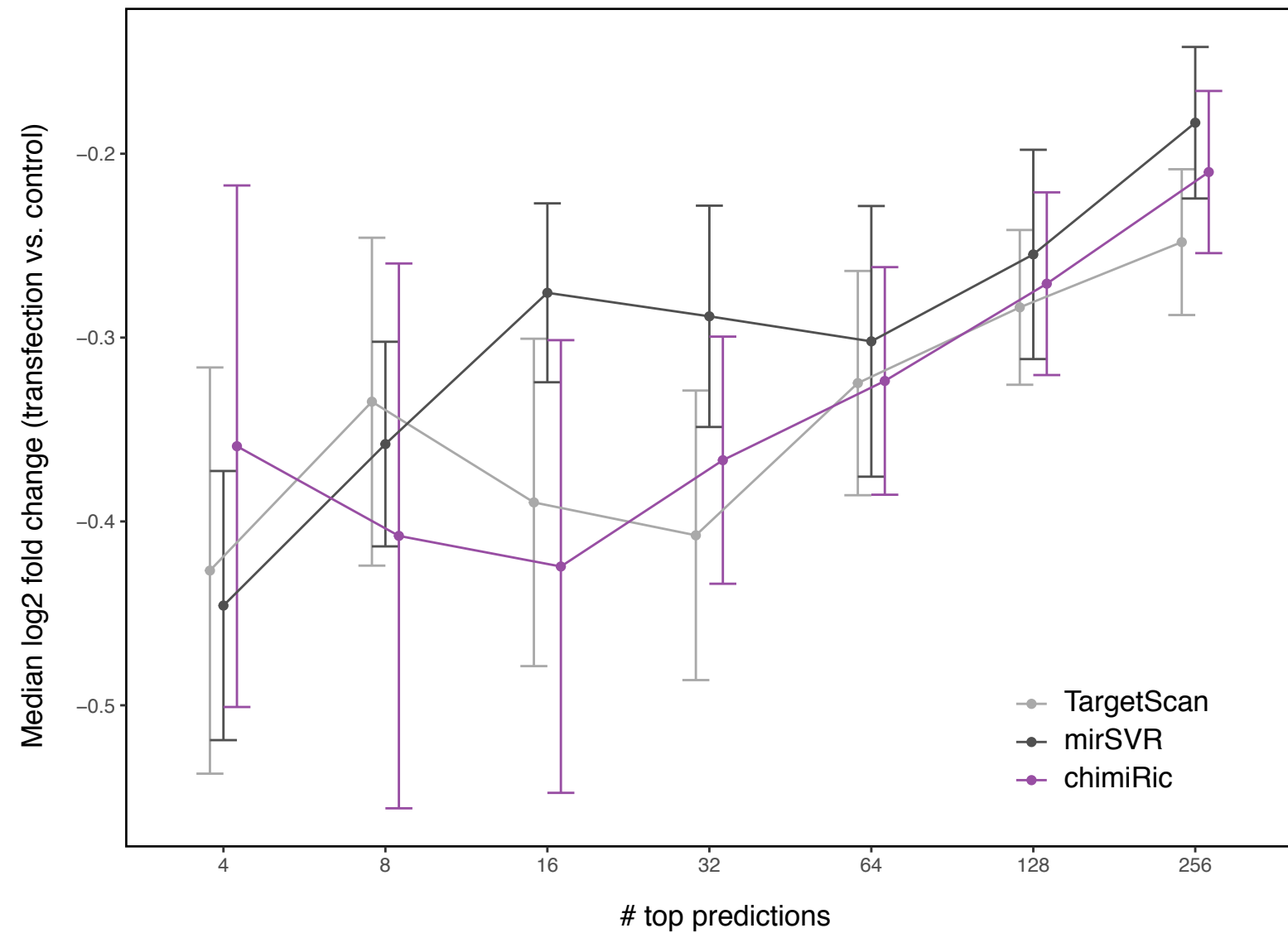

Supplement: S3 Fig — The extent of mRNA downregulation for the top N predictions of each method (chimiRic: purple; TargetScan: grey; mirSVR: black), including (A) or excluding (B) predicted 6-mer seed sites. The extent of downregulation was represented by the median log2 fold changes between transfection and control, while the variation between eight data sets was represented by standard error bars. (PDF) [file pcbi.1005026.s003.pdf]

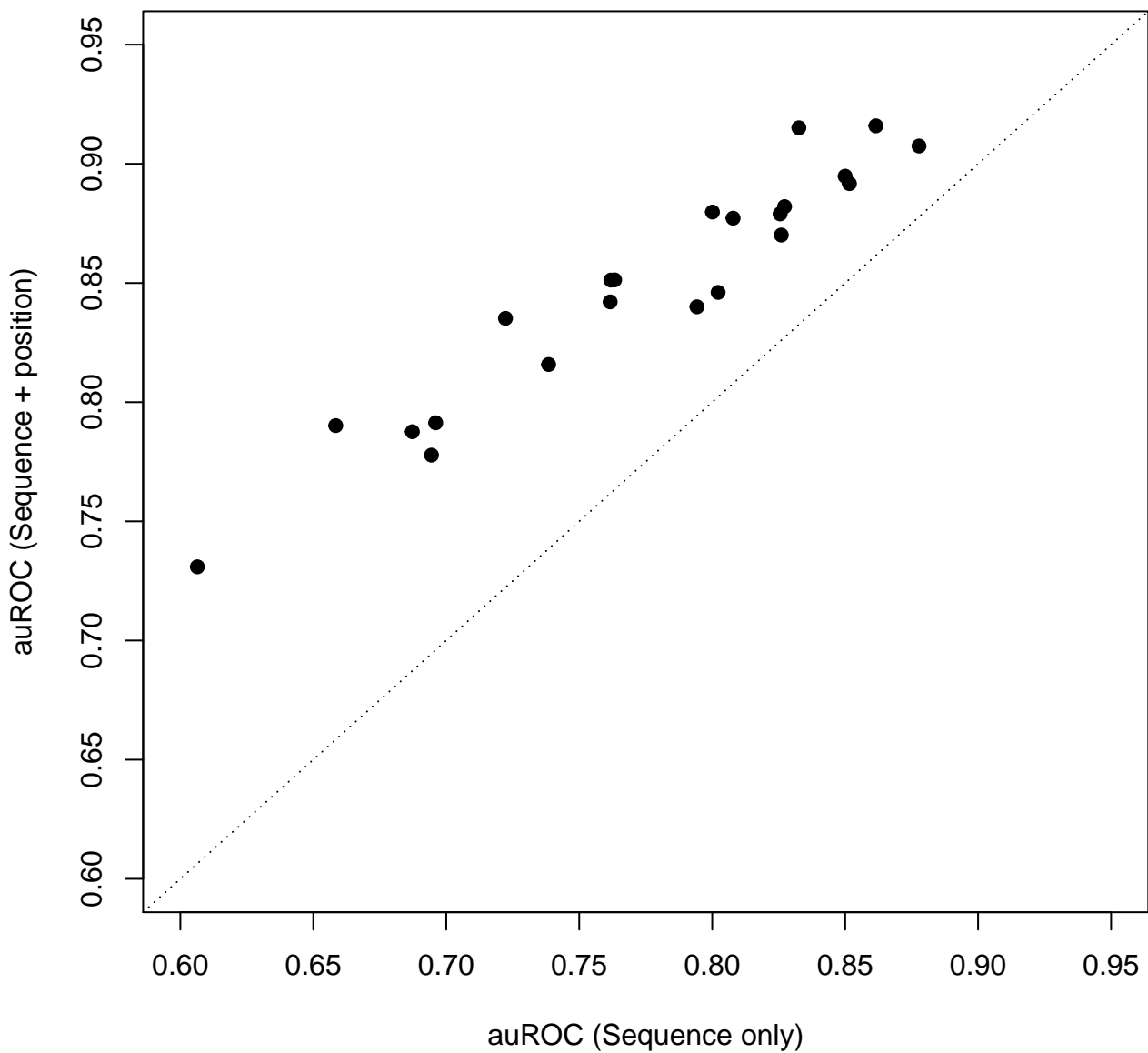

Supplement: S4 Fig — Each data point represents the performance on one held-out miRNA family in HEK293 CLIP data set, where the x-axis represents the auROC of the chimiRic AGO binding model without positional features and the y-axis represents the auROC of the full chimiRic AGO binding model. (PDF) [file pcbi.1005026.s004.pdf]

Upstream of 6-mer seed match

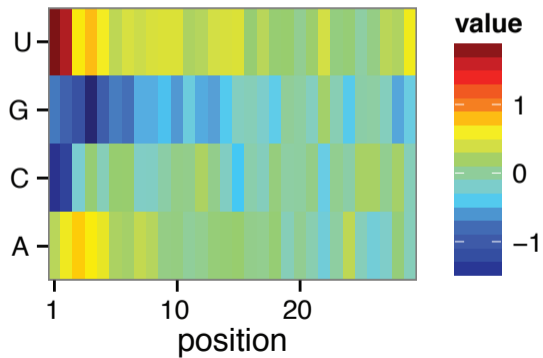

Downstream of 6-mer seed match

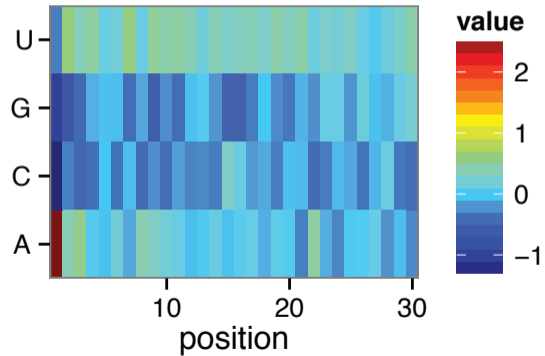

Supplement: S5 Fig — The POIMs for upstream/downstream positional 1-mer components of the common chimiRic AGO binding model are visualized as heatmaps. Position 1 in downstream and upstream sequences matches nucleotide 1 and nucleotide 8 in the miRNA, respectively. Therefore the most significant single-nucleotide features correspond to m1A and m8/9U. (PDF) [file pcbi.1005026.s005.pdf]
